# Caffeic acid methyl and ethyl esters exert potential antidiabetic effects on glucose and lipid metabolism in cultured murine insulin-sensitive cells through mechanisms implicating activation of AMPK

**DOI:** 10.1080/13880209.2017.1345952

**Published:** 2017-08-23

**Authors:** Hoda M. Eid, Farah Thong, Abir Nachar, Pierre S. Haddad

**Affiliations:** a Natural Health Products and Metabolic Diseases Laboratory, Department of Pharmacology and Physiology, Université de Montréal, Montreal, Canada;; b Canadian Institutes of Health Research Team in Aboriginal Antidiabetic Medicines, Montreal Diabetes Research Center, Montreal, Canada;; c Department of Pharmacognosy, Faculty of Pharmacy, University of Beni-Suef, Beni-Suef, Egypt;; d Faculty of Dentistry, University of Toronto, Toronto, Canada

**Keywords:** Adipogenesis, Akt, hepatic glucose output, insulin resistance, GLUT4

## Abstract

**Context:** Caffeic acid methyl (CAME) and ethyl (CAEE) esters stimulate glucose uptake and AMP-activated protein kinase (AMPK) in C2C12 myocytes (ATCC^®^ CRL-1772^TM^).

**Objective:** Effects of CAME and CAEE were now assessed on myocyte glucose transporter GLUT4 activity and expression, on hepatic gluconeogenesis and on adipogenesis as well as major underlying signaling pathways.

**Materials and methods:** GLUT4 protein translocation was studied in L6 GLUT4*myc* cells, glucose-6-phospatase (G6Pase) in H4IIE hepatocytes and adipogenesis in 3T3-L1 adipocytes. Key modulators were measured using western immunoblot. Cells were treated for 18 h with either CAME or CAEE at various concentrations (12.5–100 μM).

**Results:** Myocyte glucose uptake rose from 10.1 ± 0.5 to 18.7 ± 0.8 and 21.9 ± 1.0 pmol/min/mg protein in DMSO-, CAME- and CAEE-stimulated cells, respectively, similar to insulin (17.7 ± 1.2 pmol/min/mg protein), while GLUT4*myc* translocation increased significantly by 1.70 ± 0.18, by 1.73 ± 0.18- and by 1.95 ± 0.30-fold (relative to DMSO), following insulin, CAME and CAEE stimulation, respectively. CAME and CAEE suppressed hepatocyte G6Pase by 62.0 ± 6.9% and 62.7 ± 6.0% with IC_50_ of 45.93 and 22.64 μM, respectively, comparable to insulin (70.7 ± 2.3% inhibition). Finally, CAME and CAEE almost abrogated adipogenesis (83.3 ± 7.2% and 97.3 ± 3.0% at 100 μM; IC_50_ of 13.8 and 12.9 μM, respectively). The compounds inhibited adipogenic factors C/EBP-β and PPAR-γ and stimulated AMPK activity in the three cell-lines.

**Discussion and conclusions:** CAME and CAEE exerted antidiabetic activities in insulin-responsive cells through insulin-independent mechanisms involving AMPK and adipogenic factors.

## Introduction

Caffeic acid (CA) is found abundantly in many plants as well as in plant-derived human diet. It rarely exists in free forms but is rather found as glycosides, esters and amides (LeBlanc et al. 2012). CA derivatives have a broad spectrum of pharmacological activities ranging from antibacterial to antioxidant, anti-inflammatory, anti-hypertensive, anti-thrombotic, anticancer, immunomodulatory and neuroprotective properties (Eid et al. 2010). We isolated CA methyl ester (CAME) from *Vacciunium vitis-idaea* L. berries (Ericaceae), which was later found to be a byproduct of solvent extraction with methanol (Eid 2010). Interestingly, CAME is a potent stimulator of glucose uptake in cultured C2C12 muscle cells. Similarly, other research groups reported that CA phenethyl ester (CAPE), an active ingredient of honeybee propolis, exhibited marked antidiabetic activities in both *in vitro* and *in vivo* models (Lee et al. 2007; Celik et al. 2009). We examined 20 CA derivatives for the stimulation of glucose uptake in the same cell line. Among these compounds, CAME and CA ethyl ester (CAEE) potently enhanced glucose uptake in cultured C2C12 cells through mechanisms involving AMP-activated protein kinase (AMPK), while causing no or little toxicity (Eid et al. 2010). Therefore, these two compounds are the subject of the present study aiming to further elucidate their molecular targets in skeletal muscle as well as to evaluate their antidiabetic potential in liver and adipose tissues, two major insulin-sensitive tissues that control glucose and lipid homeostasis.

AMPK has a wide role in carbohydrate and lipid homeostasis. It acts as a metabolic gauge to restore cellular energy balance by activating catabolic pathways such as glycolysis and fatty acid oxidation and by shutting down ATP-consuming pathways, including cholesterol synthesis, lipogenesis and gluconeogenesis (Ruderman et al. 2003). In skeletal muscle, which is responsible for about 75% of postprandial glucose uptake, AMPK regulates glucose uptake through activation of GLUT4 translocation from intracellular pools to plasma membrane (DeFronzo and Tripathy 2009). Furthermore, AMPK activation during exercise or by AMPK-activator such as AICAR was reported to stimulate GLUT4 expression (McGee et al. 2008). Importantly, phosphorylation of AMPK inhibits fatty acid synthesis via the phosphorylation and inactivation of ACC (Snel et al. 2012). The latter is a main lipogenic enzyme and a potent inhibitor of mitochondrial fatty acid oxidation. In the liver, AMPK downregulates the key gluconeogenic enzymes PEPCK and G6Pase (Kim et al. 2008). Interestingly, liver is the major site of action of metformin, the first-line treatment for T2DM from the biguanide family (Kim et al. 2008). Similar anti-adipogenic and catabolic effects of AMPK is conceivable in adipose tissue under conditions of higher energy demands, dietary restriction or following treatment with pharmacological agents such as the biguanides (Bijland et al. 2013). Since preadipocyte differentiation is an energy consuming process, AMPK activation leads to inhibition of adipogenesis and decreases the expression of adipogenic factors such as PPAR-γ and C/EBPs (Bijland et al. 2013). Therefore, AMPK represents an attractive target for obesity and type 2 diabetes (T2DM) management and intervention. Since CAME and CAEE are AMPK-activators in C2C12 cells, we aimed to study the effect of these compounds on AMPK activity and its downstream effectors in different murine cell lines derived from skeletal muscle, liver and adipose tissue. In this study, we used rat L6-GLUT4*myc* and L6 wild type (WT) to study GLUT4 translocation and expression, respectively. In addition, G6Pase activity and effects on adipogenesis were assessed in rat hepatoma H4IIE cells and mouse 3T3-L1 adipocytes, respectively.

## Material and methods

### Source of CAME and CAEE

CAME and CAEE were purchased from Indofine Chemical Co. (Hillsborough, NJ).

### Cell lines and culture

Rat L6 skeletal cells WT or L6 cells transfected to stably overexpress GLUT4 harboring a myc epitope on the first exofacial loop of the transporter were provided by Dr. Amira Klip, Hospital for Sick Children, Toronto. H4IIE rat hepatoma cells (ATCC^®^ CRL1548™) and 3T3-L1 adipocytes (ATCC^®^ CL-173™) were purchased from American Type Culture Collection (ATCC) (Rockville, MD). Insulin from bovine pancreas was purchased from Sigma-Aldrich (Oakville, Canada). Other reagents were purchased from Sigma-Aldrich unless otherwise noted.

Cells were seeded into 12- or 6-well plates in media containing 0.5% antibiotics (penicillin 100 U/mL and streptomycin 100 μg/mL). L6 Cells were cultured until 70% confluence in α-minimum essential medium (αMEM) containing 10% (v/v) fetal bovine serum (FBS) then switched to αMEM medium containing 2% FBS for 5–7 days to allow differentiation into multinucleated myotubes. H4IIE cells were grown in DMEM supplemented with 10% FBS and the experiments were conducted when cells were fully confluent. 3T3-L1 preadipocytes were allowed to proliferate to confluence in DMEM medium containing 10% FBS. Two days post confluence (day 0), cells were placed in the same medium to which the adipogenic cocktail (500 μM 3-isobutyl-1-methylxanthine, 500 nM insulin and 10 μM dexamethasone) was added to induce differentiation. Two days later, cells were switched to fresh DMEM containing 10% FBS and 500 nM insulin until day 8 and the medium was replenished every 2 days. All culture media were purchased from Invitrogen Life Technologies (Burlington, Canada).

### LDH cytotoxicity assay

To determine nontoxic concentrations, cells were treated overnight (16–18 h) with 0.1% DMSO or different concentrations of either CAME or CAEE (12.5, 25, 50 and 100 μM). Cell culture media were collected separately and kept on ice. The cells were rinsed with PBS then lysed with 1% Triton X-100 in culture media for 10 min and the lysate were centrifuged at 4 °C for 10 min at 250*g*. Lactate dehydrogenase (LDH) activity in medium and in lysates was assayed with the LDH-Cytotoxicity Assay Kit II (BioVision, Mountain View, CA) according to the manufacturer's protocol (Smith et al. 2011). LDH activity was expressed as the ratio of released LDH (medium) to total (medium + lysate) LDH activity.

### ^3^H-Deoxyglucose uptake assay

Myotubes were serum-starved for 4 h prior to treatment with either vehicle (DMSO, 0.1%), CAME or CAEE (50 μM) for 18 h (unpublished time course results show maximum activity after 18 h treatment) or insulin (100 nM) for 15 min. Measurement of glucose uptake was performed as previously described (Eid et al. 2010) using [^3^H]-2-deoxyglucose (TRK-383, Amersham Biosciences, Buckinghamshire, UK). Nonspecific uptake was measured in the presence of cytochalasin B (10 μM) and was subtracted from all values.

### Determination of cell surface GLUT4 using o-phenylenediamine dihydrochloride (OPD) assay

L6-GLUT4*myc* myotubes grown in 24-well plates were serum-starved for 4 h and were incubated with either CAME or CAEE (50 μM) for 18 h or insulin (100 nM) for 15 min. Cell-surface GLUT4*myc* levels were measured by an antibody-coupled colorimetric assay (Niu et al. 2003) using *O*-phenylenediamine dihydrochloride (OPD) reagent (1 mL/well) (Sigma-Aldrich, St. Louis, MO), an anti-c-*myc* antibody (1:200 dilution; Santa Cruz Biotechnology, Santa Cruz, CA) and horseradish peroxidase (HRP)-conjugated goat anti-rabbit IgG at 4 °C (1:1000 dilution; Cell Signaling Technologies, Danvers, MA).

### Enzymatic activity of G6Pase

At 90% confluence, H4-IIE cells were treated with 12.5, 25, 50 and 100 μM of either CAME or CAEE in serum free medium. Vehicle (0.1% DMSO)-treated and 100 nM insulin-treated cells served as the negative and positive control respectively. The activity of G6Pase was assessed by measuring the rate of glucose production in the presence of a non-limiting amount of glucose-6-phosphate (G6P) as described elsewhere (Nachar et al. 2013). Total glucose production was measured using a commercial glucose assay kit (AutoKit Glucose; Wako Diagnostics, Richmond, VA). Protein content was measured using bicinchoninic acid (BCA) assay (Simpson 2008) and G6Pase activity was expressed in relation to protein content.

### Adipogenesis assay

Starting from day 0 of differentiation, 3T3-L1 adipocytes were separately treated with different concentrations of either CAME or CAEE (12.5, 25, 50 and 100 μM). Cells treated with vehicle (0.1% DMSO) in proliferation and differentiation media were used as negative controls. The adipogenic compound rosiglitazone (5 μM) was added to some vehicle-treated wells and served as the positive control. At day 8 after initiation of differentiation, intracellular lipid accumulation was quantified as previously described using AdipoRed Reagent according to the manufacturer’s protocol (Martineau et al. 2010).

### Thymidine incorporation assay

To assess for the inhibitory activity of CAME and CAEE on and mitotic clonal expansion of 3T3-L1 adipocytes, cells were separately treated with 12.5, 25, 50 and 100 μM of CAME or CAEE for 16 h in differentiation media and the incorporation of radio-labeled methyl-^3^H-thymidine (MP Biomedicals cat #240410, Irvine, CA) into the newly replicated DNA was measured as described elsewhere (Martineau et al. 2010). The incorporated radioactivity was measured in a liquid scintillation counter (LKB Wallac 1219 Rackbeta; Perkin Elmer Life and Analytical Sciences, Inc., Boston, MA).

### Western immunoblotting

L6 cells myotubes, H4IIE cells and 3T3-L1 adipocytes were treated with either CAME, CAEE (50 μM each) or vehicle (DMSO) for 18 h. Insulin (100 nM) or aminoimidazole carboxamide ribonucleotide (AICAR; 1 mM) were added to some vehicle-treated wells 30 min prior to the end of the treatment and served as the positives controls. Protein content was assayed by the bicinchoninic acid method (Thermo Scientific Pierce Protein Research, Rockford, IL) standardized to bovine serum albumin. Western immunoblotting was performed as described elsewhere (Eid et al. 2010). Membranes were incubated overnight with phospho-AMPK, phospho-Akt, GLUT4, GLUT1 and β-actin antibodies (1:1000 dilution, Cell Signalling Technology, Danvers, MA), or with PPAR-γ, SREBP-1 (1:200, Santa Cruze Biotechnology, Santa Cruz, CA), followed by incubation with HRP-coupled secondary antibody (1:10,000) for 1 h. Immunoreactive bands were visualized by enhanced chemiluminescence and quantified by the Scion Image program (Scion Corporation, Frederick, MD). Experiments were repeated on 3 different passages of cells, each passage containing all conditions in parallel.

### Statistical analysis

Data are reported as the mean ± SEM with the number of replicates and number of independent experiments indicated. Results were analyzed by one-way analysis of variance (ANOVA) with a Fisher *post-hoc* test using SPSS software, version 24 (IBM Corporation, Armonk, NY). IC_50_ values were calculated by fitting the results to standard pharmacological dose–response algorithm using PRISM software version 6 (GraphPad Software Inc., La Jolla, CA). Statistical significance was set at *p* ≤ 0.05.

## Results

### Cytotoxicity assay

Similar to our previous study in C2C12 cells, the maximum nontoxic concentrations of CAME and CAEE were determined using a LDH assay kit. Concentrations that induced less than 10% cell death were chosen as the maximum nontoxic concentrations (Table 1).

**Table 1.** Maximum non-toxic concentrations of CAME and CAEE in μM.

	Compound				
Cell line	CAME	CAEE			
L6 GLUT4*myc*	50	50			
H4IIE	100	50			
3T3-L1	100	100			

### CAME and CAEE stimulate glucose uptake and GLUT4 translocation in L6-GLUT4myc myotubes

**Figure 1.**
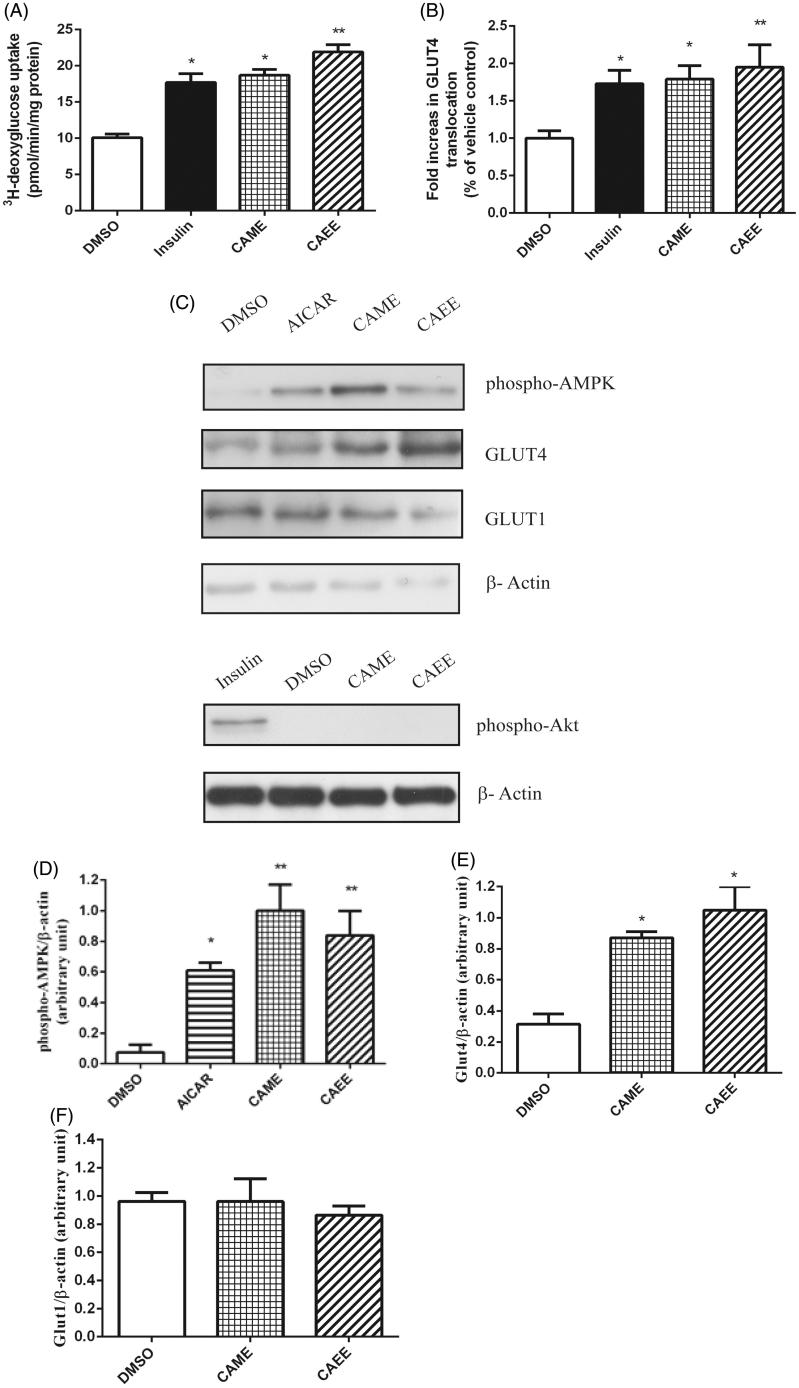
(A) CAME and CAEE increase ^3^H-deoxyglucose uptake in L6 GLUT4*myc* myotubes. Cells were treated with 50 μM of either CAME, CAEE, or with vehicle (0.1% DMSO) for 18 h. 100 nM insulin served as the positive control and was applied for the last 15 min of the treatment in vehicle-treated cells. Glucose uptake was performed as described in Materials and Methods section. (B) CAME and CAEE stimulate GLUT4 translocation in L6 GLUT4*myc* myotubes. Cells were treated as previously described, cell surface GLUT4*myc* was detected by an enzyme-linked colorimetric assay. By the end of the treatment, L6- GLUT4*myc* cells were labeled with anti-*c-myc* antibody as described in Materials and Methods section. Results represent the means ± SEM of three independent experiments, and 3-4 cells were analyzed for each condition per experiment. *Indicates a significant (*p* ≤ 0.05) difference, **(p < 0.01) from the vehicle control group as assessed by ANOVA. (C) CAME and CAEE increase phosphorylation of AMPK and protein content of GLUT4 but do not alter phospho-Akt and GLUT1 protein levels in L6 myotubes. L6 myotubes treated with CAME or CAEE (50 μM each), DMSO (0.1%) for 18 h. Insulin (100 nM) and AICAR (2 mM) both applied for 30 min and served as positive controls. Immunoblots were probed with anti phospho-AMPK (Thr 172), anti-GLUT4, anti-GLUT1 and anti-phospho-Akt (Ser 473) antibodies as described in Materials and Methods section. Anti-β-actin antibody was used as a loading control. Representative blots are shown in panel C. Data are expressed as (D) phospho-AMPK/β-actin, (E) GLUT4/β-actin and (F) GLUT1/β-actin and are given as mean ± SEM from 3 experiments. *p < 0.05 indicates a significant difference, **(*p* < 0.01) from the vehicle control group.

Under basal condition, 2-deoxyglucose uptake rate in L6-Glut4*myc* myotubes was 10.1 ± 0.5 pmol/min/mg protein. Upon 15 min treatment with insulin (positive control), glucose uptake rate was significantly stimulated to 17.7 ± 1.2 pmol/min/mg protein (175 ± 13% increase in glucose uptake as compared to DMSO), (*p* < 0.05, Figure 1(A)). Consistent with our previous observations in cultured C2C12 cells (Eid et al. 2010), CAME and CAEE significantly stimulated glucose uptake rate to 18.7 ± 0.8 and 21.9 ± 1.0 pmol/min/mg protein, respectively, which corresponds to 184 ± 16%, and 216 ± 19% increase in glucose uptake, respectively, as compared to the vehicle control (DMSO) (*p* < 0.05, Figure 1(A)). To elucidate the mechanism by which CAME and CAEE stimulated glucose uptake, we measured cell surface GLUT4*myc*. Under basal condition, the entire cohort of GLUT4*myc* is sequestered in intracellular compartments. Following stimulation with insulin or activation of AMPK, glucose uptake in skeletal muscle occurs through the translocation of GLUT4 to the cell membrane. Our data show that CAME and CAEE significantly stimulated GLUT4 translocation in L6-GLUT4*myc* muscle cells by 1.79 ± 0.18- and 1.95 ± 0.30-fold relative to DMSO, respectively (*p* < 0.05, Figure 1(B)). Insulin induced 1.73 ± 0.18-fold increase in GLUT4 translocation (*p* < 0.05, Figure 1(B)), in accordance with previously published results (Cushman et al. 1998).

### CAME and CAEE stimulate AMPK and increase GLUT4 protein content in L6 myotubes

CAME and CAEE increased AMPK phosphorylation in L6 myocytes (13- and 11-fold increase over DMSO, *p* < 0.01, Figure 1(C,D)) indicating an elevation in AMPK activity, greater than values in AICAR-treated cells (8.5-fold increase over DMSO, *p* < 0.05, Figure 1(C,D)). On the other hand, CAME and CAEE had no effect on Akt (Figure 1(C)).

Fully differentiated skeletal muscle myotubes express two forms of GLUT: GLUT4 and GLUT1, accounting for insulin-stimulated and basal glucose uptake, respectively. Hence, we wanted to know whether the increase in glucose uptake by CAME and CAEE was associated with increased GLUT4 and GLUT1 protein contents. Interestingly, both compounds increased GLUT4 content by ∼2.7- and 3.2- fold, respectively, compared to DMSO (*p* < 0.05, Figure 1(C,E)). In contrast, CAME and CAEE had no effect on GLUT1 protein levels (Figure 1(C,F)).

### CAME and CAEE inhibit G6Pase activity and stimulate AMPK in H4IIE cells

**Figure 2.**
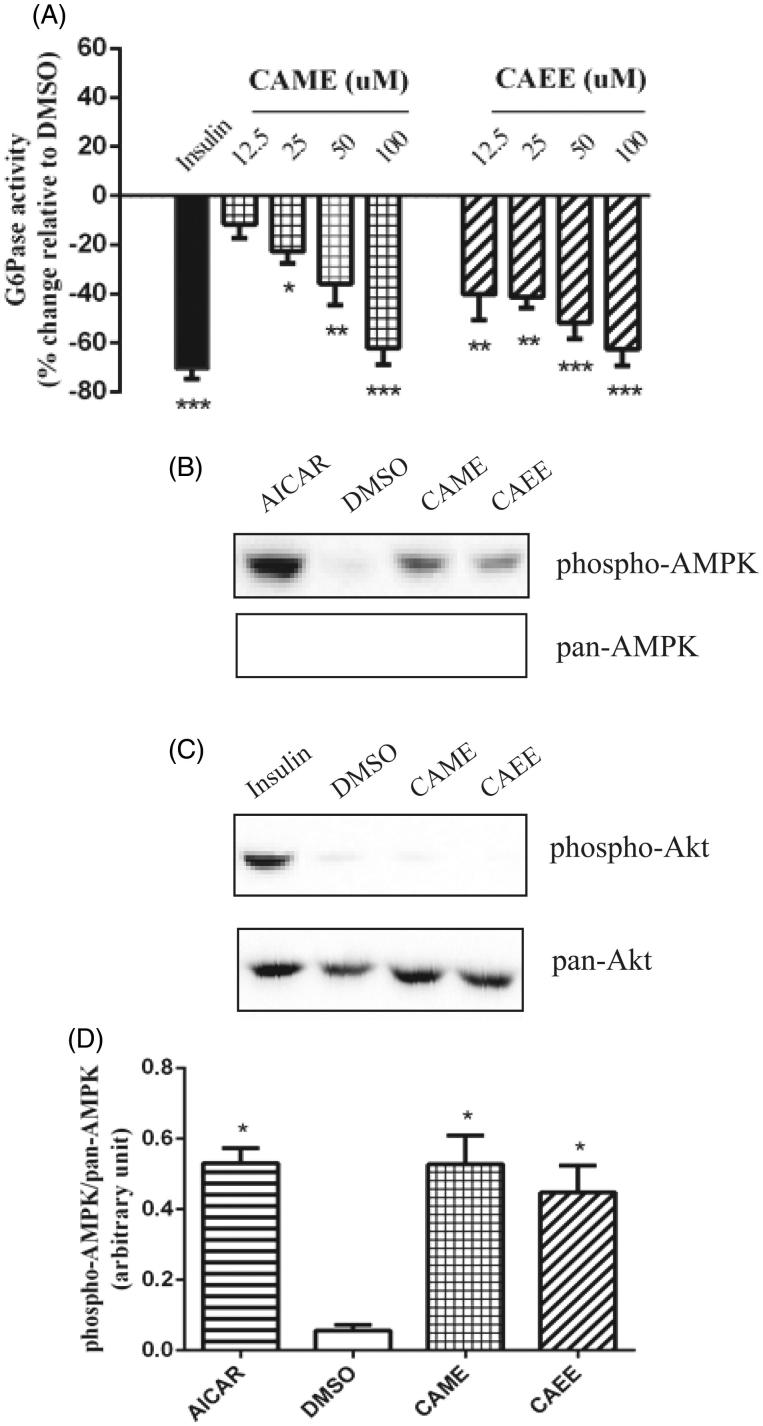
CAME and CAEE inhibit G6Pase by mechanisms involving activation of AMPK. (A) H4IIE hepatocytes were treated for 16 h with several concentrations (12.5, 25, 50 and100 μM) of either CAME or CAEE and G6Pase activity was determined colorimetrically using a commercial glucose assay kit as described under Materials and Methods. Data are expressed relative to vehicle control (0.1% DMSO, 0% inhibition). Insulin (100 nM) applied for similar treatment period served as the positive control. (B and C) Cells were treated with the optimal nontoxic concentration of either CAME, CAEE (50 μM each), or DMSO (0.1%) for 18 h, insulin (100 nM), AICAR (1 mM) applied for 30 min served as the positive controls. (B) The upper blot was probed with anti-phospho-AMPK antibody (Thr 172) and the lower blot was probed with anti-pan AMPK. (C) Blots were probed with antibodies against phospho-Akt (Ser 473) or pan-Akt. Immunoblots are representative of three independent experiments with similar results. (D) Data are expressed as phospho-AMPK/pan-AMPK and are given as mean ± SEM from 3 experiments. *p < 0.05 indicates a significant difference, **(*p* < 0.01), ***(*p* < 0.001) from the vehicle control group.

CAME and CAEE resulted in a significant inhibition of G6Pase activity that reached 62.0 ± 6.9% and 62.7 ± 6.0% at 100 μM (Figure 2(A)). IC_50_ values were 45.93 and 22.64 μM, respectively. Insulin applied for the same treatment period inhibited G6Pase activity by 70.7 ± 2.3% relative to DMSO (*p* < 0.05, Figure 2(A)). This inhibition was accompanied by an increase in AMPK phosphorylation to the same extent as the positive control AICAR (*p* < 0.05, Figure 2(B,D)). Similar to the results in L6 cells, CAME and CAEE did not increase the phosphorylation of Akt in H4IIE cells (Figure 2(C)).

### CAME and CAEE inhibit adipogenesis, proliferation, mitotic clonal expansion and stimulate AMPK in 3T3-L1 adipocytes

**Figure 3.**
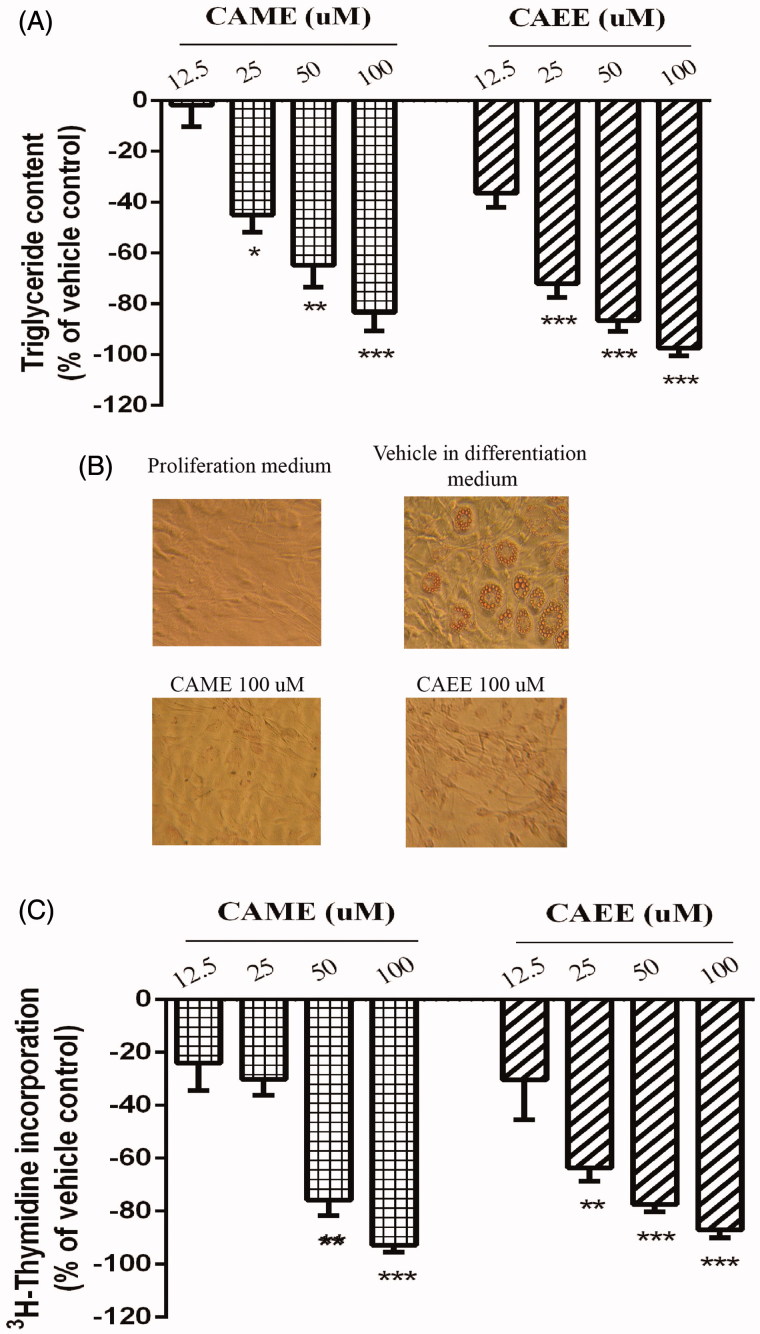
(A and B) Anti-adipogenic effect of CAME and CAEE in 3T3-L1 adipocytes. Cells were treated with several concentrations (12.5, 25, 50 and100 μM) of either CAME or CAEE in DMEM containing 10% FBS and the standard adipogenic cocktail (500 μM 3-isobutyl-1-methylxanthine, 500 nM insulin and 10 μM dexamethasone) for 2 days then in DMEM containing 10% FBS and 500 nM insulin starting from days 2-8 of differentiation. This medium was changed every 2 days. (A) On day 8, lipid content was measured by AdipoRed assay. Results represent the means ± SEM of three independent experiments. *Indicates a significant (*p* ≤ 0.05) difference, **(*p* < 0.01), ***(*p* < 0.001) from the vehicle control group. (B) Phase-contrast micrographs at 200X of AdipoRed-stained cells on day 8 demonstrate adipocyte morphology. Vehicle-treated cells show the presence of lipid droplets. On the other hand, cells treated with the maximum nontoxic concentration (100 μM) of either CAME or CAEE retained the fibroblast-like morphology of pre-adipocytes and were devoid of lipid droplets. (C) CAME and CAEE inhibit pre-adipocyte clonal expansion. Confluent 3T3-L1 pre-adipocytes undergoing clonal expansion in response to differentiation cues were treated with vehicle or 12.5, 25, 50 and 100 μM of either CAME or CAEE for 20 h and incorporation of ^3^H-labeled thymidine was measured. Normalized data are presented as mean ± SEM of three independent experiments. **Indicates a significant (*p* ≤ 0.01) difference, ***(*p* < 0.001) from the vehicle control group.

**Figure 4.**
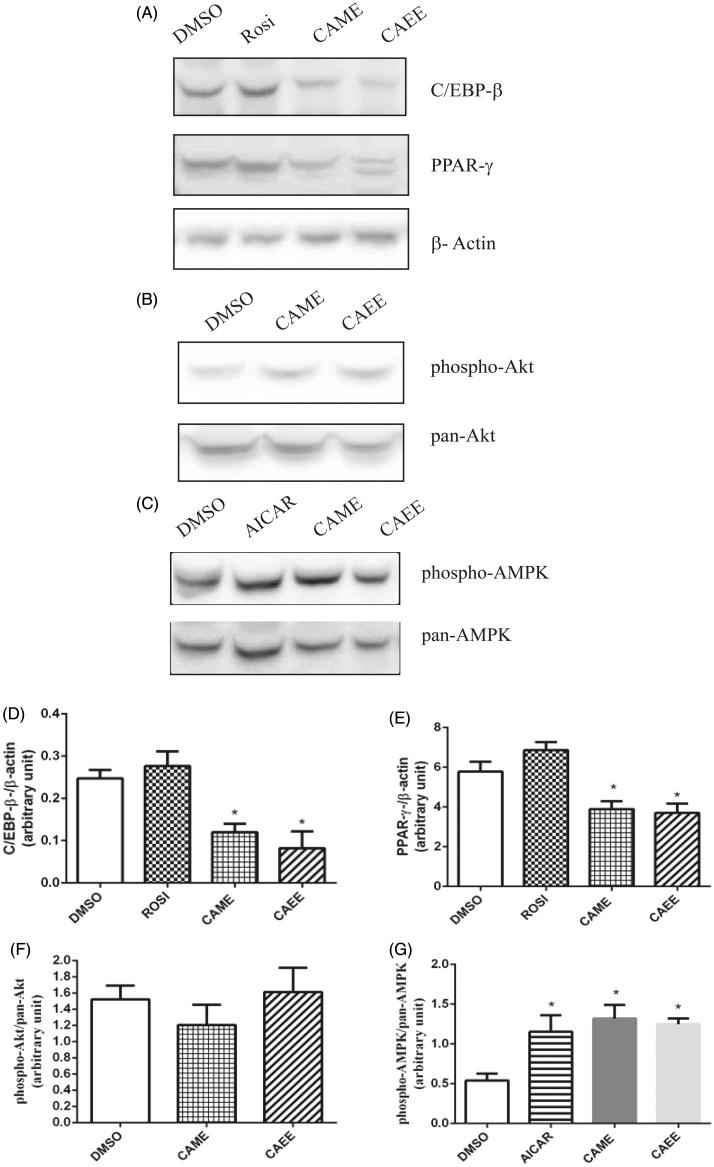
CAME and CAEE inhibit C/EBP-β and PPAR-γ protein expression through mechanisms implicating AMPK pathway. Shown are representative blots of 3T3-adipocytes treated with the maximum nontoxic concentration (100 μM) of either CAME or CAEE. Blots are probed with antibodies against C/EBP-β, PPAR-γ, β-actin (A), phospho-Akt and pan-Akt (B) or phospho-AMPK and pan-AMPK (C). All values are mean ± SE from three independent experiments. *Indicates a significant (*p* ≤ 0.05) difference from the vehicle control group. Data are expressed as C/EBP-β/β-actin (D), PPAR-γ/β-actin (E), phospho-Akt/pan-Akt (F) and phospho-AMPK/pan-AMPK (G). Rosi: rosiglitazone.

We first investigated the effect of CAME and CAEE on adipogenesis in 3T3-L1 adipocytes by measuring the accumulation of intracellular lipid droplets at day 8 of differentiation. As shown in Figure 3(A), the two compounds markedly and significantly inhibited adipogenesis. At 100 μM, CAME and CAEE almost completely blocked lipid accumulation (inhibition by 83.3 ± 7.2% and 97.3 ± 3.0%, respectively, Figure 3(A), *p* < 0.001) with IC_50_ of 13.8 and 12.9 μM, respectively. Adipocyte differentiation in such conditions was comparable to that of non-differentiated control cells. Micrographs show retention of fibroblast-like morphology of pre-adipocytes with no evidence of lipid droplet accumulation at 100 μM concentrations (Figure 3(B)). To test if the anti-adipogenic effect of the two compounds occurs early during adipogenesis, 2-days-postconfluent cells were treated with 12.5, 25, 50 and 100 μM of either CAME and CAEE in differentiation medium and cell proliferation was measured. CAME and CAEE significantly inhibited thymidine incorporation into 3T3-L1 cells undergoing mitotic clonal expansion (inhibition reached 92.7 ± 2.6% and 86.9 ± 3.0% at 100 μM of CAME and CAEE, respectively, *p* < 0.001), IC_50_ values were 39.4 and 13.1 μM, respectively, suggesting an early inhibition of adipogenesis (Figure 3(C)). In parallel, the protein levels of C/EBP-β and PPAR-γ, the key early adipogenic factors, were significantly reduced (*p* < 0.05, Figure 4(A,D,E)). Our results thus suggest that CAME and CAEE inhibited adipogenesis by suppressing the early adipogenic events and adipogenic factors. Next, we examined the effect of CAME and CAEE on insulin and AMPK pathways, the key regulators of adipocyte metabolism and adipogenesis. CAME and CAEE did not modulate Akt phosphorylation (Figure 4(B,F)) but significantly activated AMPK measured as phospho-AMPK (2.4- and 2.3-fold increase over basal levels, *p* < 0.05, Figure 4(C,G)).

## Discussion

Obesity and diabetes have reached epidemic proportions worldwide. According to a recent World Health Organization (WHO) report, the number of overweight individuals increased to 2 billion in 2014, 600 million of them are clinically obese (WHO 2015), while ∼387 million adults live with diabetes across the globe according to the International Diabetes Federation (IDF 2014).

Obesity-induced insulin resistance in skeletal muscle, liver and adipose tissue lies at the core of a group of metabolic abnormalities known as the metabolic syndrome that, when clustering together, increase the risk for developing T2DM and cardiovascular diseases (Roberts et al. 2013). In skeletal muscle, insulin resistance is manifested as impaired insulin-stimulated glucose transport. Glucose cannot diffuse across the lipid bilayer of cell membrane and therefore needs a particular group of transporters called GLUT. GLUT4 is the main insulin-responsive transporter and is located primarily in skeletal muscle cells, cardiac muscle cells and adipocytes, where it is responsible for most of the glucose uptake (James et al. 1989; Verhey et al. 1995). In the basal state, the majority of GLUT4 is retained in intracellular storage compartments. Binding of insulin to its receptor activates the phosphatidylinositol 3-kinase (PI3K)-Akt pathway and triggers a sequence of events that ultimately leads to the translocation of GLUT4 to the plasma membrane and stimulation of cellular glucose uptake (Cushman et al. 1998). The second important pathway regulating glucose uptake is activated during exercise and involves the stimulation of GLUT4 translocation by a mechanism that implicates AMPK (Osler and Zierath 2008; Mungai et al. 2011). In addition to GLUT4, GLUT1 is also expressed in skeletal muscle and is found to be responsible for basal glucose uptake. Unlike GLUT4, which is expressed in insulin-sensitive tissues, GLUT1 is ubiquitously distributed and resides on the plasma membrane under the basal state (Pascual et al. 2004).

The present study aimed to elucidate the mechanism by which CAME and CAEE exert their glucose uptake stimulating activity in skeletal muscle cells in culture. The effects of these two compounds on GLUT4 were studied in L6 skeletal muscle cells expressing GLUT4 with an exofacial myc epitope affording the opportunity to study GLUT4 translocation (Cushman et al. 1998). In addition, these cells express more of GLUT4 than the C2C12 cell model we previously used (Tortorella and Pilch 2002). We also investigated the effects of the two caffeic acid derivatives on GLUT4 and GLUT1 total protein content. Our results demonstrated that CAME and CAEE potently stimulated glucose uptake in L6 cells after 18 h treatment. This increase surpassed that obtained in our previous study in C2C12 cells (Eid et al. 2010) possibly due to greater GLUT4 protein content in L6 cells. This effect was accompanied by increased GLUT4 translocation and expression showing that GLUT4 plays an important role in mediating the effect of CAME and CAEE on glucose uptake in L6 skeletal muscle cells. Unlike that of GLUT4, GLUT1 protein level remained unaffected by the treatment with either CAME or CAEE. Similar to our previous results in C2C12 cells (Eid et al. 2010), the stimulatory effects of CAME and CAEE on glucose uptake and GLUT4 translocation did not involve insulin-dependent pathways, but was rather mediated by phosphorylation and activation of AMPK.

In obesity, increased free fatty acid influx into liver impairs insulin signaling and leads to increased hepatic gluconeogenesis. This contributes to elevated fasting blood glucose levels in T2DM patients (Jung and Choi 2014). Importantly, metformin, the most widely prescribed antidiabetic drug, exerts its antihyperglycemic action mainly through the reduction of hepatic gluconeogenesis (Viollet et al. 2012). It is believed that AMPK activation mediates the action of metformin on the gluconeogenic enzymes (Kim et al. 2008). Since G6Pase is a central enzyme of hepatic gluconeogenesis, pharmacological treatments aiming to restore signaling pathways regulating the expression and enzyme activity of G6Pase are regarded as potential treatment solutions for T2D. Therefore, we studied the effect of CAME and CAEE on the activity of this enzyme. Both were found to inhibit G6Pase. In an attempt to elucidate the mechanism of G6Pase inhibitory activity, the effects of the two caffeic acid esters on insulin signaling and AMPK activity in H4IIE cells were also investigated. In agreement with our results in the other cell models, CAME and CAEE activate AMPK in H4IIE cells by stimulating its phosphorylation without inducing changes in Akt phosphorylation.

Despite the growing obesity epidemic, present therapeutic options remain limited in number and effectiveness (Kang and Park 2012). Therefore, novel safe and effective anti-obesity drugs are needed. Inhibition of adipogenesis constitutes a potential therapeutic target for the management of obesity (Flach and Bennett 2010). Adipogenesis can be targeted via different mechanisms including PPAR-γ antagonism and activation of AMPK kinase (Martineau et al. 2010). In this study, we investigated the effect of CAME and CAEE on adipocyte differentiation in 3T3-L1 cells, a well-established cell model to study adipogenesis. The process of adipocyte differentiation follows a complex transcriptional cascade and occurs in two phases: mitotic clonal expansion (MCE) and terminal differentiation (Bijland et al. 2013). In this study, we report that treatment of 3T3-L1 preadipocytes with either CAME or CAEE during the early phase of differentiation reduced the accumulation of lipid droplet. The two compounds interfered with adipogenesis starting at MCE and reduced the expression of the early adipogenic factor C/EBP-β and its target PPAR-γ, the master regulator of adipogenesis (Ruderman et al. 2003). This was accompanied by an increase in the activity of AMPK but no change in Akt. Therefore, we suggest that the effect of CAME and CAEE on 3T3-L1 adipogenesis are mediated by mechanisms involving the activation of AMPK. Despite the fact that the link between AMPK activation and downregulation of adipogenic factors is well established (Gao et al. 2008), further studies are needed to test whether AMPK inactivation by compound C or AMPKα siRNA treatment will restore adipogenesis and expression of adipogenic transcription factors.

## Conclusions

**Figure 5.**
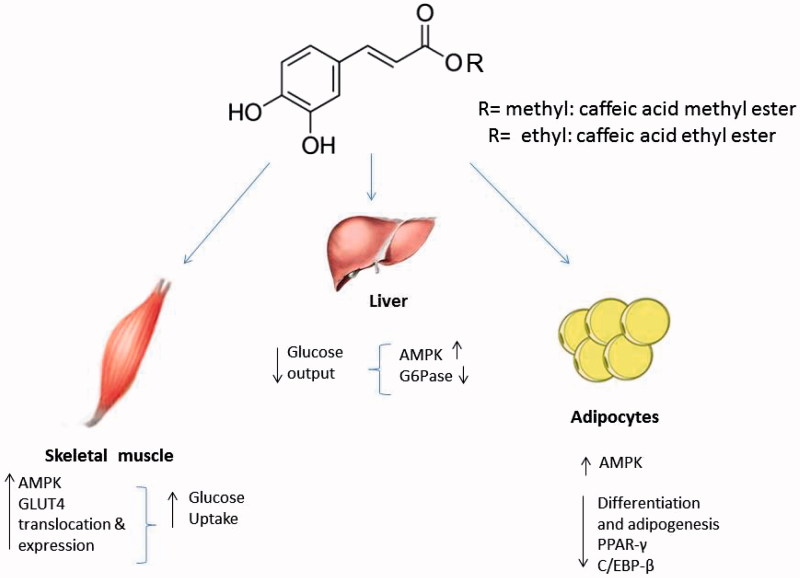
A proposed model for biological effects and signaling pathways modulated by caffeic acid methy ester (CAME) and caffeic acid ethyl ester (CAEE) *in vitro*.

In summary, CAME and CAEE are able to potently stimulate glucose transport in skeletal muscle, reduce hepatocellular glucose production and adipogenesis in an AMPK-dependent manner (see schematic representation in Figure 5). The results presented here provide evidence that caffeic acid derivatives represent a class of compounds with a promising antidiabetic and anti-obesity potential and warrant further study to explore the detailed mechanisms of action of these compounds.
